# Can an early selection system accelerate the maturity of candidates of both genders in medical school?

**DOI:** 10.5116/ijme.5856.6d72

**Published:** 2017-01-08

**Authors:** Jerome Rene Lechien, Chantal Kempenaers, Michele Dramaix, Paul Linkowski

**Affiliations:** 1 CIUM ASBL, Comite InterUniversitaire des étudiants en Médecine, University of Mons (UMONS), Belgium; 2 Psychiatry Department, Erasme Hospital, ULB Medical School, Brussels, Belgium; 3 Biostatistics Department, ULB Medical School, Brussels, Belgium

**Keywords:** Gender, medical school, selection, success, Belgium

## Introduction

Academic success at university depends on many variables, including high school education, social and economic background, cultural origins and behavioural elements.^1^^,^^2^ Factors for medical school success include intrinsic motivation, intelligence quotient, emotional quotient, regularity of work, sense of self-efficacy, maturity and creativity.^3^^,^^4^ The organisation and the admission systems of medical schools also have an impact on academic success. Thus, in some Western countries such as France and Belgium, medical studies often have various selection systems, either before students begin their studies or after the students’ first year of study.^5^^-^^8^ The debate still continues about the time and content of the selection process, and the impact of gender on success.^5^^,^^9^ Some believe that an entry selection process before admission, based on the basic sciences, is the best solution. Others are in favour of a selection process that takes place after a year, or more of study and that is based on the subjects taught to students. The medical school system in the French Community of Belgium (FCB) has a predefined number of courses accounting for an ‘academic year’, with a total of seven academic years. To be able to progress onto the following academic year, students must pass all courses with an average grade of 12/20. If unsuccessful, students must repeat the academic year and complete the courses that they failed the previous year. Also, ever since 1997 the health care system in Belgium has imposed a national selection process to access medical resident training. As such, there is a primary selection process before or during medical school that corresponds to the number of medical resident places available upon graduation. At first, between 1997 and 2003, the FCB established a primary selection system at the end of the third year of study. Students then began their medical curriculum at the end of the third year, and only a predefined number could continue this curriculum. Students who failed the selection process could repeat the third year to be ranked higher the following year. All students who passed the selection system were guaranteed to have a place on the course and a place to study a specialisation. Based on information about international medical courses, it appears that the FCB is the only region in the world to implement a delayed selection process at the end of the third year of medicine. Since the end of the 1990s, women have increasingly entered FCB medical schools. Thus, over the past few decades, European medical studies have seen a significant feminisation of the profession. Certain studies have observed a more stable trajectory for female than for male students.^5^^,^^10^ Generally, these studies have reported that female students have a more positive attitude towards academic work than male students. It is suggested that male students are less engaged in their studies, meaning that they also adapt less to the university environment.^5^ Also, women are reported to be more efficient regarding non-cognitive skills (empathy and communication), which can be predictive of success at medical school.^9^

The selection process could further emphasise the adaptive advantage that female students have.^5^ This theory, however, remains controversial, as many studies have shown no significant differences in the adaptation to university between male and female students.^11^ We conducted a recent analysis of the trajectory of students according to gender using two groups of students. The first group was composed of students that were not subject to a selection process. The students of the second group were subject to a selection process at the end of the third academic year. Students were ranked according to their marks obtained during the first three years at university. The selection system consisted of the selection of a specified number of students who were then allowed to continue the medical curriculum.

### From success in the first three years of success in the last year

In 2002 a first study showed that there was a significant relationship between the marks obtained at the end of the curriculum and the marks of the third year. This study was based on a group of 102 students who were not subject to an early or delayed selection process.^8^ Based on their grades, this study reported that student progress was not very stable. Three-quarters of the students either progressed or regressed by the end of the curriculum compared to their position in the first three years of study.^8^ In 2006 a second study was carried out on a group of 82 students who went through the third year selection process. It showed that a significant amount of the marks obtained at the end of the curriculum could be explained by those obtained in the first academic year.^7^ This suggests the difference in students' behaviour when faced with a selection filter.^7^ Based on these findings, a retrospective examination was carried out on the impact of gender on the students’ level of success. It was found that the extent to which the first year marks predicted those obtained at the end of the curriculum was more pronounced for male than for female students. Moreover, linear regression showed a significant predictability of the sixth year marks based on the second year marks, for male and female students, when using the selection system after the third year. The impact of marks after three years on those after six years was negative in the group of students who did not undergo the selection process and positive in the group that did undergo the selection process.

Based on the analysis of a selection system at the end of the third academic year, these observations do not quite corroborate the data in the literature showing a more stable trajectory for female than for male students.^10^ Bearing in mind that the selection process increases competition, some authors have shown that female students have higher anxiety than male students and are more motivated to achieve their goals. As for the male students, they perceive failure as a loss of personal value and are more likely to avoid any risk of failure unless they are particularly confident or intrinsically motivated. Fear of failure, tendency to procrastinate and high self-confidence seem to be typical characteristics of most male students. However, anxiety and the value attributed to performance could be higher among female students.^5^ The masculine battle is a cognitive one and can have two outcomes: i) success if the student is sufficiently motivated and confident, ii) failure and dropping out of the course if the demands seem too high. This is in contrast to the scenario of female students since they believe, often wrongly, that they are incompetent and need to succeed to realise that they are in fact capable.^5^ These theoretical differences could explain the results, which show a reduction in the difference between female and male students when using the marks after the selection process to predict their performance in the sixth year. Indeed, it can be postulated that the second year male students have already undergone a first selection process, simply related to the success of the first year, and that they represent a group of motivated and relatively confident students. On the contrary, the implementation of selection process impacts female students more psychologically is catalysing their anxiety and their daily work pressures as they strive for early success. However, caution is still required as the selection process studied did not take place early in the curriculum but at the end of the third year. On the other hand, a bias that may occur is that professors could assess students who were subject to a selection process differently compared to students who were not.

If a moderate link between first year success and success at the end of the sixth year does exist, an early selection system could accelerate the maturity of many candidates. This could help students to adapt more quickly to the academic environment and quickly adapt their final way of working. Unlike an entrance exam, this filter could give the opportunity to all students to adapt to university teaching. Efficient systems are in place to help students to adopt their final way of working, including remediation courses and mentoring systems.^4^ Further studies are needed to confirm the impact of gender on success when students are subject to a selection system or not.

### Acknowledgements

The authors would like to thank Amy Gorton and Natalie Dickson, native English speakers for proofreading of the manuscript.

### Conflict of Interest

The authors declare that they have no conflict of interest.
